# Parametric Study towards Optimization of a Short Duration Carbonation Process of Recycled Cement Paste

**DOI:** 10.3390/ma15196513

**Published:** 2022-09-20

**Authors:** André Silva, Rita Nogueira, Alexandre Bogas, Dariusz Wawrzyńczak, Aleksandra Ściubidło, Izabela Majchrzak-Kucęba

**Affiliations:** 1 Civil Engineering Research and Innovation for Sustainability, Department of Civil Engineering, Instituto Superior Técnico, Universidade de Lisboa, Avenue Rovisco Pais, 1049-001 Lisboa, Portugal; 2 Department of Advanced Energy Technologies, Faculty of Infrastructure and Environment, Czestochowa University of Technology, Dabrowskiego 73, 42-201 Czestochowa, Poland

**Keywords:** carbon capture utilization and storage, short carbonation process, industrial viability, CO_2_ uptake, recycled cement paste powder

## Abstract

The recycling process of concrete originates a byproduct, cement paste powder (CPP), which is a material composed mainly of hydrated cement. This cementitious material has demonstrated promising results when applied as a binder in new concrete batches, provided it has been subjected to a previous carbonation process. One of the obstacles to the industrial application of this strategy is the long duration of the typical carbonation process, which requires from 3 to 28 days. Recently, the authors have developed a short two-hour carbonation process and thoroughly analysed it over its entire extension. In this paper, a parametric analysis of the carbonation process is performed towards CO_2_ uptake maximization, aiming to increase the feasibility of its short duration. CO_2_ uptake is evaluated using the ignition by furnace method and thermogravimetric analysis. Among the parameters considered, the initial water content and the CPP thickness present the highest impact on CO_2_ uptake. The investigation of different CO_2_ concentrations inside the carbonation chamber showed that the maximum CO_2_ uptake does not occur for the highest concentration value. Moreover, a minimum resident time for the forced carbonation of CPP in industrial contexts is presented, and is found to be highly dependent on the CO_2_ concentration. The particle size and purity degree of CPP revealed a limited influence on the CO_2_ uptake achieved. Additionally, this paper provides further insight into the mechanisms involved in the carbonation of mature cement paste while increasing the feasibility of our recently proposed short duration carbonation process.

## 1. Introduction

Concrete is estimated to be responsible for about 5–8% of total CO_2_ emissions annually, driving significant environmental problems due to the huge magnitude of consumption of this construction material throughout the world [1,2] Even though concrete is composed of several components, cement remains the major contributor to its environmental impact, being responsible for 80–95 wt.% of the total carbon emissions [3,4,5].

Tackling this environmental issue, recent investigations have targeted two technologies identified by the Cement Technology Roadmap as having major potential for effective reduction of carbon emission in the cement industry, and consequently in the concrete industry: (1) carbon capture utilization and storage (CCUS) and (2) clinker substitution [6,7,8,9,10]. These studies exploit the carbonation capacity of mature concrete at the end of its life cycle while simultaneously targeting its potential reactivity as a binding component in new concrete batches. The cementitious material addressed in these studies is a fully hydrated cement paste powder (CPP), which is a byproduct of the recycling process of mature concrete at the end of its life cycle [11,12].

Another environmental issue connected with the construction industry sector is the huge annual production of construction and demolition waste (CDW), estimated at 35–46% [13,14] of total waste stream generation in the European Union. The main outlet for this waste is dumping and landfilling, which includes end-of-life concrete, responsible for around 12–40% of this CDW [14,15].

To overcome this negative impact and promote circular economies, investigations focused on the utilization of recycled aggregates in cementitious mixtures have been progressively developed [14,15,16]. Even though the objective of the recycling process is to obtain recycled aggregate for further concrete batches, it returns about 10–20% of a byproduct composed of CPP and aggregate impurities [11,12]. Note that a method to obtain only CPP from this byproduct has already been studied by Carriço et al. [17], who developed a separation method that allows CPP to be retrieved from concrete waste with a purity of nearly 90 vol.%. Nevertheless, considering the novelty of this line of investigation, the authors resorted to synthetic lab-made cement pastes.

Different studies have investigated the application of CPP as a supplementary cementitious material (SCM) in mortars; however, it is only with the introduction of a carbonation process that performance results have demonstrated a promising prospect [6,7]. The main focus was the performance of cementitious mixtures adopting carbonated CPP as an SCM, wherein CPP was exposed to CO_2_ during 12 to 28 days; hence, the details of the carbonation process were not discussed in detail, which was identified as a gap that the current paper intends to cover. Nevertheless, the carbonation of hydrated cementitious materials has been studied in other works, which reported that CO_2_ reacts with several components present in CPP, namely, calcium silicate hydrate (C-S-H), calcium hydroxide (CH), and in lower percentages, ettringite (AFt), monosulfate (AFm) aluminate, calcium carbonate ($C\bar{C}$), and anhydrous cement particles [9,18,19]. Note that the two main calcium-bearing compounds present in CPP, C-S-H and CH, show different carbonation mechanisms. In C-S-H, CaO is removed from the C-S-H structure, reducing the C/S ratio of the compound following Equation (1) and originating a mixture of decalcified C-S-H, $C\bar{C}$, and free water. On the other hand, CH reacts with CO_2_, resulting in $C\bar{C}$, free water, and heat, following Equation (2) [20,21,22].

(1) $${\left(\mathrm{CaO}\right)}_{x+0.67}{\mathrm{SiO}}_{2}{\left(H_{2}O\right)}_{y}+{\mathrm{xCO}}_{2}\to {\left(\mathrm{CaO}\right)}_{0.67}{\mathrm{SiO}}_{2}{\left(H_{2}O\right)}_{y-z}+{\mathrm{xCaCO}}_{3}+{\mathrm{zH}}_{2}O$$

(2) $$H_{2}{\mathrm{CO}}_{3}\left(\mathrm{aq}\right)+\mathrm{Ca}{\left(\mathrm{OH}\right)}_{2}\left(s\right)\to {\mathrm{CaCO}}_{3}\left(s\right)+2H_{2}O\;+74\;\mathrm{kJ}/\mathrm{mol}$$

Despite this, the long duration of the carbonation process usually applied, ranging between 12 and 28 days, hampers the implementation of an industrially viable process. To tackle this problem, a short-term carbonation process with a duration of two hours and a CO_2_ concentration of 80% was developed and thoroughly analysed in its entire extension in our previous paper [23]. The setup parameters defined there allowed for a significant carbonation degree (47.9%), especially considering the low duration of the process. However, there is room for optimizing the parameters to maximize CO_2_ uptake and improve the feasibility of the process. The previous paper clarified the different carbonation rates of the diverse compounds present in CPP during the two-hour period. While most CPP compounds react quickly with CO_2_ within the first few minutes (e.g., the Ca(OH)_2_ or CH reaches a carbonation degree of 85% in 18 min), the carbonation rate of calcium silicate hydrate (C-S-H) is much slower, reaching a carbonation degree of 33% by the end of the process. Thus, the investigation of the carbonation process parameters that influence the amount of CO_2_ uptake by CPP during the process is important to reach a better understanding of the conditions that promote acceleration of the carbonation reaction kinetics. Ultimately, this paper aims to refine the carbonation process parameters, thereby maximizing the CO_2_ uptake and increasing the feasibility of its industrial application in the concrete industry.

Following the carbonation process setup defined in the previous paper, the following parameters were considered for the parametric analysis: CPP initial water content (IWC); CO_2_ concentration (CO_2_c) inside the carbonation chamber; CPP maximum particle size (PS); CPP layer thickness (LT); and CPP powder type (PT). Taking into account the purpose of maximizing CO_2_ uptake by CPP, the ignition by furnace method (IFM) [24] was used along with thermogravimetric analysis (TGA). These techniques enable assessment of the variation in the water content of the CPP throughout the process, which was carried out in order to explore the relationship between this feature and CO_2_ uptake.

## 2. Materials and Methods

### 2.1. Materials

Portland cement (OPC)-CEM I 42.5R with a mineralogical composition of C3S = 56.7%, C2S = 16.6%, C3A = 10.0%, C4AF = 9.2% was used in this study. The OPC presented a specific surface (Blaine) of 452 m2/kg and a density of 3070 kg/m3, with a chemical composition of CaO = 62.76%, SiO_2_ = 19.42%, Al_2_O_3_ = 5.39%, Fe_2_O_3_ = 3.00%, MgO= 1.74%, K_2_O = 0.53%, Na_2_O = 0.12%.

Lab-made CPP originating from cement paste (CPPp) was produced from a well-hydrated cement paste with a water/cement ratio of 0.45, which was cured in a moist chamber for 1 year. Additionally, CPP originating from concrete (CPPc) was produced and cured in similar conditions to the cement paste using a standard concrete composition, as described in Table 1.

The CPPp and CPPc specimens were then dried at 100 °C for 2 days, crushed in a jaw crusher, and submitted to an additional drying period of 2 days to ensure maximum free water removal, which is essential to minimizing the adhesion of material to the walls of the mill in the next step. Finally, the ensuing crushed material was grinded using a jaw crusher, a roller mill, and a ball mill followed by sieving through 250 µm mesh. After this process, the water content of CPP was 1.5 wt.%.

A specific procedure was developed to achieve CPP samples with different initial water content in order to study the influence of this parameter on carbonation. The process started by mixing CPP with water at a ratio of 1:1, obtaining a very fluid inert paste. This paste was then spread in a tray, ensuring a very small thickness of material, and placed in a furnace at 80 °C to promote the evaporation of free water. The CPP was removed from the furnace after the time required to achieve a given water content. These periods were determined from a previous experiment in which the mass loss evolution of CPP inside the furnace was measured in periodic and constant time intervals. Wet CPP presented a looser appearance and homogeneous water content across the CPP layer.

### 2.2. Carbonation Methods

**Figure 1:**
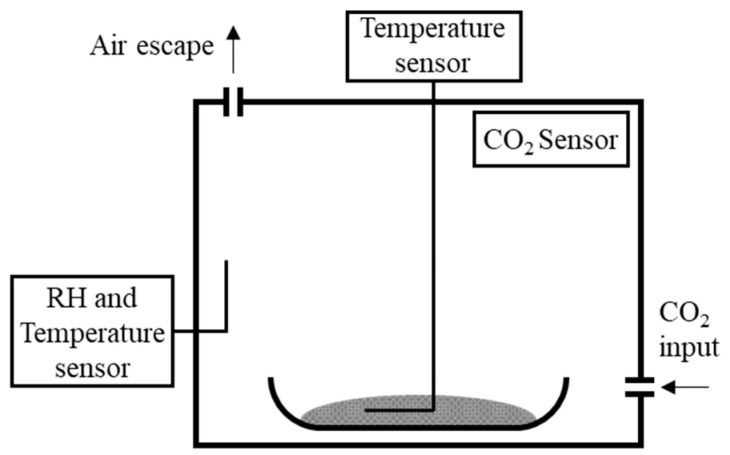
Equipment setup used in the carbonation test.

The carbonation process used in each parametric analysis was performed using an airtight chamber (Figure 1) with a volume of ≈87 liters. In each carbonation test, 100 g of CPP were exposed to the pre-defined CO_2_ concentration (CO_2_c) for 2 h. CO_2_c was monitored over time, as well as the RH, temperature of the atmosphere (initially set at 60% and 20 °C, respectively), and temperature of the CPP.

For selection of the parameters (IWC, CO_2_c and PT), the effect of carbonation was investigated throughout the entire extension of the two-hour period. Conversely, for the remaining parameters (PS and LT), the characterization was carried out only after ending the two-hour period.

**Figure 2:**
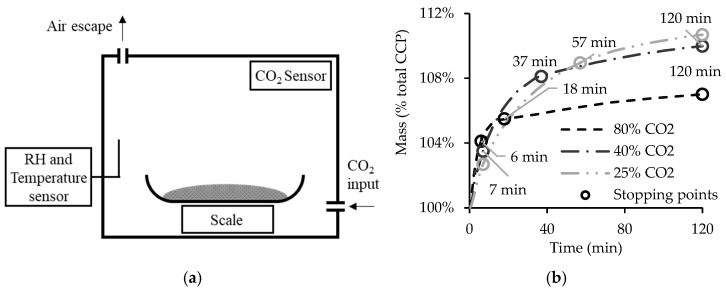
Equipment setup for the mass growth analysis (**a**) and the mass growth of CPP and stopping points considered during the carbonation process (**b**).

For the former characterization, two repetitions of the carbonation test were carried out with a shorter duration in order to collect CPP samples over time for subsequent testing. The carbonation period of these additional tests was defined with the aim of obtaining a good representation of the evolution of the carbonation reaction, which was inferred from the growth of the CPP mass. For this purpose, the previous setup was adapted (Figure 2a) to enable the evaluation of the mass growth rate of CPP during the 2 h test. Hence, three stopping points were defined (1SP, 2SP, and 3SP) for three different rates of mass growth (rapid, moderate, and slow), as shown in Figure 2b [23].

Considering that CO_2_c is expected to affect the evolution of the carbonation reaction, the repetitions were carried out for each CO_2_c to assess the corresponding stopping points connected with the aforementioned rates of mass growth (rapid, moderate, and slow). Hence, 1SP and 2SP had different durations for each CO_2_c: 1SP varies from 6 to 7 min and 2SP from 18 to 57 min. Finally, the last stopping point (3SP) has the duration settled on for the process, 120 min. Note that the option of using stopping points with the same duration regardless of the CO_2_ concentration would have hampered accurate analysis of the different reaction kinetics, as the points that mark significant alterations in the reactions would have been discarded.

Table 2 summarizes the parametric analysis performed in this paper, indicating the diverse testing conditions adopted for each parameter. The section in which each parameter was analysed as well as the corresponding testing techniques adopted are presented as well. Table 2 shows the different SPs used for each parameter under analysis depending on the data required to reach conclusions in each case. Finally, it is worth mentioning that CPPp is referred to throughout the paper as CPP, except in the section related to the CPP powder type analysis, where CPPp is compared with CPPc.

### 2.3. Characterization Methods

TGA was carried out on a NETZSCH STA 449F3 Thermobalance (Berlin, Germany). Each sample (around 60 mg) was heated at a rate of 10 °C/min in a He-gas flowing atmosphere until a maximum temperature of 1000 °C was reached. The temperature ranges of each mass loss were determined using the stepwise method. For correct comparison between results, each sample was normalized to the non-ignited component using the loss on ignition value (LOI).

IFM [24] was used as a simple, effective and inexpensive procedure for assessing the CO_2_ uptake of CPP before and after the carbonation process to complement TGA. Following the same theoretical principles as TGA, 10 g of the CPP sample were heated to 550 °C and maintained at this temperature for about 1 h, after which the mass was recorded. Then, the sample was heated to 950 °C following the same procedure as before, and the relative CO_2_ uptake was calculated according to Equation (3). Similarly, the free water content of CPP was calculated by heating a sample to 100 °C for 24 h.

(3) $${\mathrm{CO}}_{2}\mathrm{uptake}\;\left(\%\right)=\frac{{\mathrm{Mass}}_{\mathrm{at}\;550\;℃}-{\mathrm{Mass}}_{\mathrm{at}\;950\;℃}}{{\mathrm{Mass}}_{\mathrm{at}\;20\;℃}}\times \frac{1}{\left(1-\mathrm{LOI}\right)}$$

## 3. Results and Discussion

### 3.1. Influence of Initial Water Content

**Figure 3:**
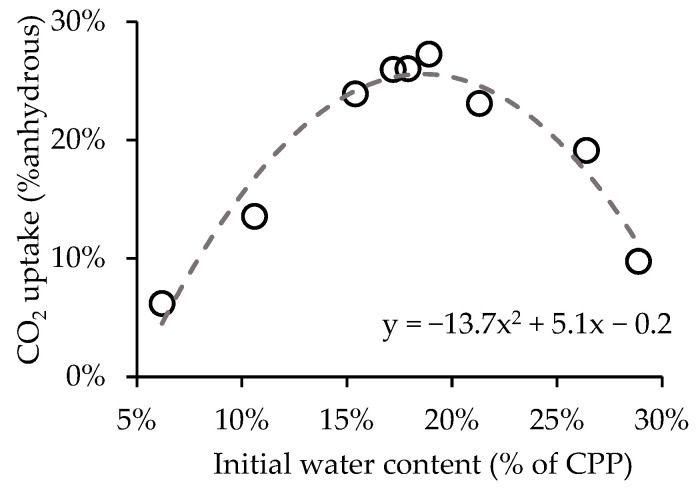
CO_2_ uptake of CPP after 1 h for different initial water content.

Figure 3 shows the influence of initial water content on CO_2_ uptake obtained for a 1 h carbonation period and CO_2_ concentration of 80%. The initial water content ranges from 5% to 30%, enabling significant differences in the CO_2_ uptake. The best performance was obtained for 19% water content (CO_2_ uptake of 27%), an intermediate value between the tested limits, which can be explained by the impact of the air/water ratio inside the porous space between the powder particles. Carbonation requires both CO_2_ dissolution and CO_2_ diffusion, which are mechanisms that increase when water varies in opposite ways, as too much water hinders the latter condition [25]. Conversely, very low CO_2_ uptake values were obtained for the lowest (6%) and the highest (29%) water content (6% and 10%, respectively).

A polynomial equation was adjusted to the data in Figure 3 to identify the initial water content value associated with the maximum CO_2_ uptake. We obtained an R2 of 0.95, indicating a good adjustment of the equation to the data. The derivative of the fitted curve indicated 18.5% as the optimum initial water content value, returning a CO_2_ uptake of 25.5%. However, for practical reasons, a wider range around this value was established for CPP carbonation. This is relevant because the CPP initial water content has to be increased artificially by water addition, as previously explained. Hence, in this case, an optimal initial water content between 16% and 21% (18.5% ± 2.5%) was established for CPP carbonation, returning a CO_2_ uptake not lower than 25% per the equation of the fitted curve. This result was applied in all of the tests reported in this work (Table 2). Note that in CPPc the water content may be lower and proportional to the cement fraction in concrete waste material. Moreover, CPP with 17% initial water content returns an RH of 88% in the atmosphere near the surface.

### 3.2. Influence of CO_2_ Concentration

**Figure 4:**
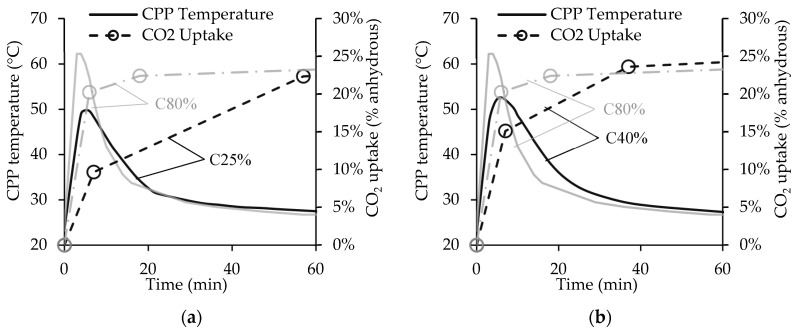
CO_2_ uptake and temperature for 25% and 40% CO_2_ concentration. Comparison between C25% and C80% (**a**) and between C40% and C80% (**b**).

This Section analyses the influence of the CO_2_ concentration inside the chamber’s atmosphere (CO_2_c) on CO_2_ uptake and CPP temperature. Figure 4 presents the first hour of the carbonation tests for C25%, C40%, and C80% (standing for CO_2_c of 25%, 40%, and 80%, respectively). All the curves present near-steady behaviour during the second hour; for this reason, this period was not included. C25% and C40% are included in Figure 4a,b, respectively, along with the C80% curves [23] in both figures (grey lines) for easier comparison. The results indicate similar behaviour for each CO_2_ concentration, with a much higher carbonation rate phase before 2SP than after this point, as previously observed for C80%. As expected, the carbonation rate before 1SP decreases with the CO_2_ concentration, presenting CO_2_ uptake values at this point of 10%, 15%, and 20% for C25%, C40%, and C80%, respectively. However, by the time process reaches 2SP, the three CO_2_ concentrations present similar CO_2_ uptake values, between 22% and 24%, suggesting a carbonation threshold at this point. This result can be explained by the depletion of calcium-bearing compounds other than C-S-H in CPP, promoting deceleration of the carbonation reaction rate [23]. In fact, after 2SP, among these calcium bearing compounds only C-S-H remains active in the carbonation reaction. Therefore, this point defines the minimum necessary resident time for the waste material under forced carbonation of a continuous industrial process. Note that from 2SP until the end of the process, C25% and C80% present a CO_2_ uptake rate of 0.020%/min, while C40% presents a slightly higher value of 0.026%/min. The different CO_2_ concentrations existing in the chamber had little influence on the carbonation rate of C-S-H, and, therefore the CO_2_ uptake, after 2SP. At the end of the carbonation test, C40% reported the highest CO_2_ uptake at 25.8%, followed by C80% and C25% with 24.5% and 23.7%, respectively. Even though C40% presented the best carbonation performance, the variation between C40% and the worst performer (C25%) was only 2.1%. This small difference should be considered when assessing the impact on energy efficiency of the carbonation process in an industrial context, especially if flue gas is considered as a prospective source of CO_2_.

In order to further test the efficiency of this carbonation process and the impact of the CO_2_ concentration, a second test with an atmospheric CO_2_ concentration (0.04%) was conducted for two weeks, with CO_2_ uptake measured after each week. The ultimate CO_2_ uptake obtained was very similar to that obtained in the two-hour process, at 26.0%, a difference of around 1.3% on average. However, only 16.3% had been achieved after one week, suggesting that the rapid/moderate growth carbonation stage was ongoing during the second week, highlighting the high impact of the CO_2_ concentration on this stage of the process.

Similar to the CO_2_ uptake, the CPP temperature presents generic behaviour during the carbonation process. The results show a quick and elevated burst of heat at the beginning following the high initial carbonation rate, then a reduction in the CPP temperature, which remains constant until the end. Comparing the maximum heat peaks, Figure 4 indicates that this parameter is directly related to the CO_2_ concentration (the higher the concentration, the higher the peak). Note that the heat burst is mainly associated with the carbonation reaction of CH, the second highest calcium-bearing compound in CPP (wt.%) [23]; thus, a higher heat peak is associated with a higher CH carbonation rate. Hence, the temperature results suggest that C80% has the highest CH carbonation degree at 1SP, while C25% presents the lowest. This condition indicates that a lower CO_2_ concentration promotes slower carbonation of CH, as expected. Finally, both C40% and C25% present a similar descending temperature rate, which is lower than that in C80%. Following previous considerations, the slower carbonation of CH presented by C40% and C25% caused a release of heat over a longer time period and which was associated with smaller temperature peaks. Conversely, C80% showed quick consumption of CH followed by lethargic CH carbonation, and consequently a shorter heat release time.

**Figure 5:**
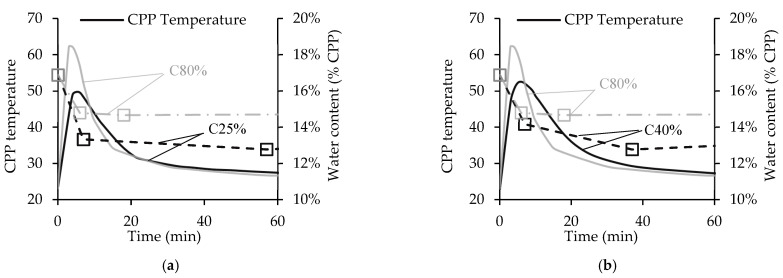
Water content and temperature for 25% and 40% CO_2_ concentration. Comparison between C25% and C80% (**a**) and C40% and C80% (**b**).

Figure 5 is similar to Figure 4, although showing the water content with CPP temperature. The three tested CO_2_c samples presented similar behaviour throughout the carbonation process, displaying a sharp water content reduction in the first few minutes (before 1SP). After this drop, water reduction was not significant, especially in the second phase of the carbonation process (after 2SP). Results suggest a close relationship between the variation of the water content and the CPP temperature, both in terms of temperature peak (and rising rate) and in terms of temperature descending rate. This is visible in the smoothest descending rate of C40%, which can explain the progressive water content reduction between 1SP and 2SP instead of the water content stabilization shown in C80%. Moreover, the relationship between water content (Figure 5) and CO_2_ uptake (Figure 4) is only visible until 1SP (the higher the CO_2_ uptake, the higher the water content); after this point, another mechanism seems to apply, as the water content tends to stabilize at a higher value in C80% (14.8%) than in C25% and C40% (13.5% and 13.3%, respectively).

The actual water content in CPP is a balance between two opposite conditions arising from the carbonation reaction of CH, that is, the water obtained as a carbonation product and the water removal caused by evaporation; this balance is very relevant due to heat release. Considering that the airtight chamber has a limited volume and rigid walls and is located in a room at ambient temperature, the water evaporation is responsible for the increase in RH, which can eventually hamper additional water evaporation. In fact, intense condensation on the chamber walls was observed for C80% from the first minute of the carbonation process. The higher the heat release, the quicker the RH increase and the higher the evaporation restriction, which is pointed out here as a possible reason for the high water content in C80%. When the initial CH carbonation burst ends (1SP), no significant heat release is produced and the water content remains relatively high, which again is because of the high amount of produced water. In C25%, both the water produced from the carbonation reaction and the initial heat release are smaller than for other CO_2_c. Therefore, the evaporation of water occurs with less restriction. In C40%, an intermediate situation occurs, with competition between the two opposite conditions: the water production prevails close to 1SP, as does the evaporation restriction (RH increment); water evaporation prevails near 2SP, where the CPP temperature has already dropped.

**Figure 6:**
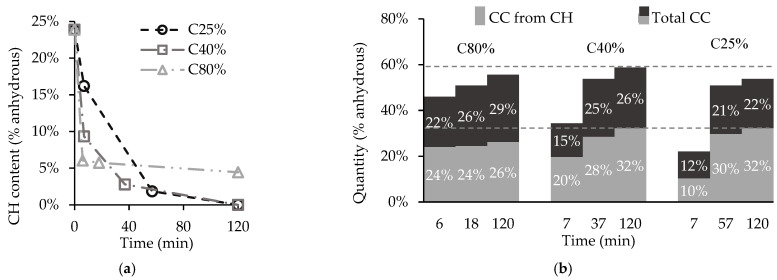
CH content consumption (**a**) and $C\bar{C}$ obtained from CH and total $C\bar{C}$ (**b**) for different CO_2_ concentrations.

Figure 6 presents the CH content for each CO_2_ concentration (Figure 6a) and the discrimination between the $C\bar{C}$ obtained from CH and from other calcium-bearing compounds (Figure 6b) using data provided by TGA. These results show that both C40% and C25% are able to consume all of the CH, unlike C80%, which was unsuccessful in carbonating all the existing CH. Moreover, Figure 6a shows that for C80%, the $C\bar{C}$ originating from CH remains nearly constant after 1SP, indicating that almost all of the carbon-accessible CH is consumed within the first 6 min. This very rapid and intense reaction supports the high heat burst at the beginning as well as the formation of a dense $C\bar{C}$ coating around the CH particles. This is commonly called the $C\bar{C}$ shield effect, which blocks further carbonation of CH [19,26,27]. Conversely, for C40% and C25% the CH is able to carbonate throughout the process (Figure 6), leading to longer heat release (Figure 5), which is continuously reduced by thermodynamic equilibrium with the lower chamber temperature. Thus, for lower CO_2_c, these results suggest a possible higher precipitation of $C\bar{C}$ away from the CH particle surface and the consequent reduction of the blocking effect caused by the dense $C\bar{C}$ coating. This result reveals a positive prospect for more complete CH carbonation in CO_2_ poorer atmospheres.

The long-term content of carbonate products is similar in all three scenarios, indicating that the amount of carbonated C-S-H is reduced for C40% and C25% (Figure 6b). Note that even though C40% and C25% were able to fully consume CH during the carbonation process, in C40% 55% of the total $C\bar{C}$ produced originates from CH, while in C25% this value is 60%. As consequence, C25% presents a lower carbonation degree (23.7% versus 25.8% in C40%). Contrarily, in C80% the incomplete CH carbonation limited the total $C\bar{C}$ produced from CH to 47%, suggesting more extensive carbonation of C-S-H.

**Figure 7:**
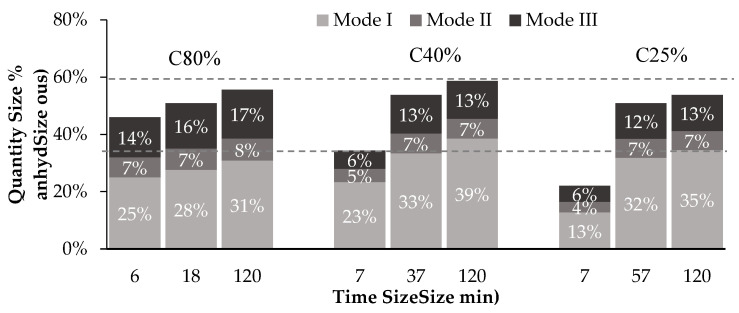
Different $C\bar{C}$ modes (I, II and III) present in each CO_2_ concentration test.

Figure 7 presents a semi-quantitative estimate of the various $C\bar{C}$ modes for different CO_2_c. These results correspond to the three progressively increasing carbonation levels that can be identified in the peak of decarbonation of $C\bar{C}$, visible in the differential thermal analysis curve of carbonated CPP between 500 °C to 800 °C [23]. Each level is usually associated with different $C\bar{C}$ polymorphs [28,29,30]: decomposition of well-crystalized $C\bar{C}$, namely, calcite, for mode I; metastable phases of $C\bar{C}$, specifically aragonite and vaterite, for mode II; and amorphous $C\bar{C}$ (ACC) for mode III.

Figure 7 points out the presence of these different polymorphs of $C\bar{C}$ regardless of the CO_2_ concentration from the beginning of the carbonation process. The formation of other $C\bar{C}$ polymorphs in addition to calcite, is usually associated with the carbonation conditions, namely, exposure to high CO_2_ content [31,32,33]. However, in previous work, the authors proposed a relationship between the calcium-bearing precursor in CPP and the $C\bar{C}$ polymorph [23], namely, $C\bar{C}$ from Mode I and Mode III, as obtained from CH and C-S-H, respectively. $C\bar{C}$ from Mode II (vaterite and aragonite) was associated with the carbonation of C-S-H, with the amount depending on the carbonation degree of C-S.H. This work further confirms these findings, as all three modes are present in significant amounts among the different CO_2_c.

A comparison between Figure 6b and Figure 7 confirms that $C\bar{C}$ obtained from CH is primarily related with $C\bar{C}$ from Mode I for the three CO_2_c, as discussed below. In C40% and C25%, $C\bar{C}$ from Mode I is always slightly higher than $C\bar{C}$ from CH (differences between 2% and 7%), $C\bar{C}$ from CH increases over time following the progressive carbonation of CH (Figure 6a), and the less stable $C\bar{C}$ compounds (e.g., aragonite, vaterite and ACC) are prone to progressive crystallization into the highly crystalline $C\bar{C}$ detected in Mode I [31,34], justifying the previously mentioned difference. Regarding C80%, the behaviour is similar with the exception of the stabilization of $C\bar{C}$ from CH after 1SP, which is explained by the corresponding trend presented by CH (Figure 6a). These results point to the high degree of crystallinity (and thermodynamic stability) in $C\bar{C}$ obtained from CH.

Additionally, the highest amount of $C\bar{C}$ from Modes II and III in C80% confirms the higher consumption of other calcium-bearing compounds, namely, C-S-H, in this CO_2_c than in C40% and C25%, as previously mentioned.

To sum up, during the two-hour carbonation process, C40% enabled a slightly higher CO_2_ uptake than C80% (and naturally higher than C25%), suggesting that the theorized shield effect of the dense $C\bar{C}$ layer expected at high CO_2_ concentrations punishes the carbonation behaviour of cementitious materials such as CPP. Yet, despite the incomplete carbonation of CH for C80%, this condition was able to promote a more extensive carbonation of C-S-H. The higher temperatures and water content in CPP caused by the shorter and more intense carbonation of CH can possibly explain this behaviour, as pointed out in other works [35]. Thus, besides the CO_2_ concentration, other factors such as moisture and temperature might have affected the CPP carbonation. Nevertheless, the results revealed low variation in CO_2_ uptake between C40% and C25%, promoting a feasible prospect for the utilization of flue gas as a source of CO_2_.

However, it should be noted that the CO_2_ concentration inside the chamber has a high impact on the duration of the first phase of the carbonation process (high/moderate carbonation rate, until 2SP), regardless of the similar CO_2_ uptake values for the different CO_2_c after the two-hour process. This conclusion highlights a minimum resident time for the forced carbonation of CPP in an industrial context that is dependent on the CO_2_ concentration, whereas the second phase (low carbonation rate, after 2SP) is considered negligible and discarded.

### 3.3. Influence of Particle Size

**Figure 8:**
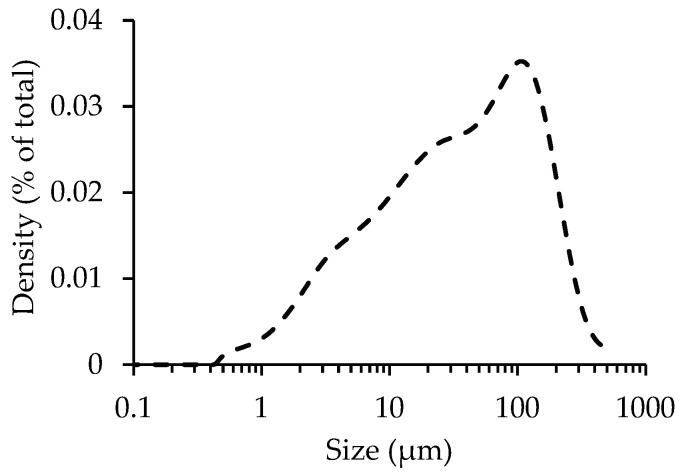
Particle size distribution of CPP before carbonation.

CPP obtained from the previously described crushing process was sieved through a 75 µm mesh, and the sample thus obtained (with all particles smaller than 75 µm) was subjected to carbonation in order to evaluate the influence of the maximum particle size on the CO_2_ uptake. The particle size distribution of CPP before sieving shows that 90% and 63% of the particles are smaller than 250 µm and 75 µm, respectively (Figure 8).

**Figure 9:**
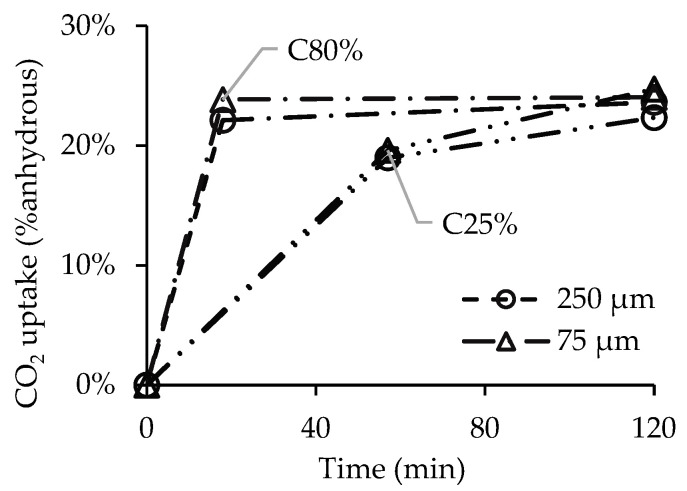
CO_2_ uptake over time for different CPP particle sizes for CO_2_ concentrations of 80% and 25%.

Figure 9 shows the CO_2_ uptake during the carbonation process for C80% and C25% for the different particle sizes. A preliminary analysis reveals that the results are seemingly similar, as reduction in the maximum particle size presents a limited effect on the enhancement of the CPP carbonation degree after the two-hour carbonation process. The carbonation rate is high until 2SP and much slower after that point, while CO_2_ uptake stabilizes between 22–25%.

Nevertheless, a more detailed analysis shows that the lowest CO_2_ concentration (C25%) was able to take more advantage of the particle size reduction, as CPP reached a CO_2_ uptake higher than that of the 250 µm size (24.7% versus 22.3%) and even slightly higher than the values obtained for C80% (24%). This result suggests that the higher specific surface of PS < 75 µm improves the ability of the lower concentration CO_2_ flux to reach more calcium bearing compounds of CPP (e.g., CH, AFt or C-S-H). On the contrary, for C80%, the higher amount of CO_2_ available did not benefit from this size reduction, possibly due to the aforementioned shield effect, preventing essential contact between the reactants.

Overall, the influence of the maximum particle size variation on CO_2_ uptake was limited, with a maximum difference of 2.4% for C25%. Furthermore, considering the impact of introducing a grinding and sieving stage on the energy cost of the CPP carbonation process, this variation becomes rather negligible.

### 3.4. Influence of Powder Thickness

**Figure 10:**
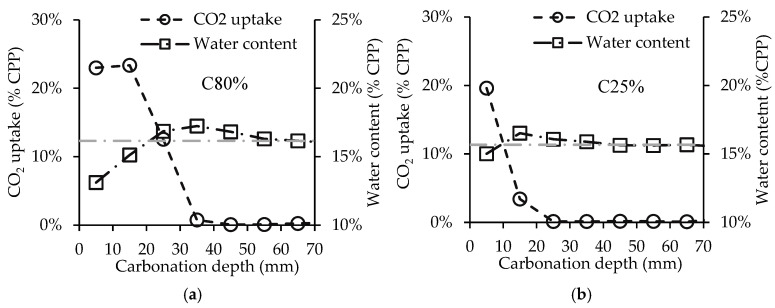
CO_2_ uptake after 2 h over carbonation depth for 80% CO_2_ (**a**) and 25% CO_2_ (**b**) concentrations.

Figure 10 presents the in-depth carbonation of a 70 mm thick CPP powder layer for C80% (a) and C25% (b) and the corresponding water content inside the sample. In the two-hour carbonation test, C80% was able to carbonate 15 mm of CPP, while C25% was limited to the first 5 mm. This result is significant, as C80% was able to carbonate twice the depth of C25%, thus allowing for higher carbonation efficiency.

Focusing on the water content, this parameter presents a slight increase in the CPP layers at the start of carbonation at 35 mm and 15 mm depth for C80% and C25%, respectively. In the CPP layers where the carbonation reaction has already occurred (until 15 mm and 5 mm in C80% and C25%, respectively) the water content shows a reduction in relation to the initial content, a reaction mechanism already reported as a result of water evaporation due to the release of heat. Note that in C80% the water content is lower near the outer surface (e.g., 5 mm depth presents a lower water content than 10 mm) even though the CO_2_ uptake is similar, which is further justified by the continuous exposure to heat release from the lower layers. In addition to this phenomenon, there seems to be another water movement towards the inward layers of the uncarbonated CPP. As a consequence, the water content increases in the layer underneath the last one that was carbonated. This increment, which may be explained by the action of gravity on liquid water produced from carbonation, is very small (<1%), and does not alter the water content of CPP to values outside the previously identified optimal range (16–21%).

### 3.5. Influence of the Amount of CPP in Concrete Waste

**Figure 11:**
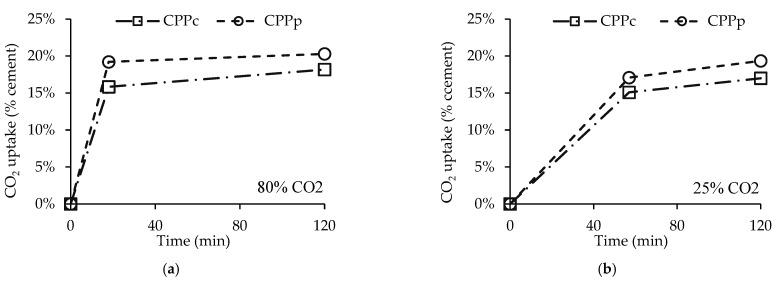
CO_2_ uptake over time for CPPc and CPPp for CO_2_ carbonation of 80% (**a**) and 25% (**b**).

Figure 11 presents C80% (a) and C25% (b) tests for two different materials: (1) CPPp (that is, the previous CPP), originating from fully hydrated cement paste, representing a high purity byproduct of a concrete recycling facility equipped with sophisticated separation treatment; and (2) CPPc, originating from fully hydrated concrete, representing a byproduct of a recycling facility without any separation treatment. Previous studies in our research centre have shown that it is possible to obtain CPP retrieved from concrete waste with up to 90 vol.% purity [17].

Here, the CO_2_ uptake values reached 2.8 g of CO_2_ per g of CPPc against 14 g of CO_2_ per g of CPPp, which is explained by the lower proportion of cement present in the former material. However, the results are closer if CO_2_ uptake is computed in terms of the initial cement mass (Figure 11). In this situation, the materials achieved nearly similar results. In fact, changing from CPPp to CPPc caused a reduction in CO_2_ uptake of only 2.1% and 2.3% for C80% and C25%, respectively, at the end of the carbonation test. This is a good result when considering that, in terms of calcium-bearing compounds, CPPc contains only 15% cement (wt.), opposed to 69 wt.% in CPPp, and that the paste composition in CPPp and CPPc has only small differences. This indicates that while CO_2_ uptake per cement weight is little affected by the composition of CPP, an efficient separation method for concrete waste may significantly increase the amount of CO_2_ uptake per amount of treated material.

## 4. Conclusions

This paper investigates five different carbonation process parameters with the objective of refining and optimizing a short carbonation process recently proposed in our previous paper, thereby increasing its industrial feasibility. Focusing mainly on the CO_2_ uptake of cement paste powder (CPP) during the carbonation process, this paper analyses the influence of its initial water content, CO_2_ concentration inside the carbonation chamber, CPP maximum particle size, layer thickness, and powder purity.

The first parameter, initial water content, revealed a significant impact on the carbonation degree of CPP, with a CO_2_ uptake difference of about 20% between the limit scenarios, uncovering a simple and efficient method of potentiating the carbonation process.

Contrarily, the CO_2_ concentration revealed a more limited influence on the carbonation degree. The CO_2_ uptake values were similar at the end of the carbonation process regardless of the CO_2_ concentration, uncovering a feasible prospect for the utilization of flue gas as a source of CO_2_. Moreover, the lowest CO_2_ concentrations (C40% and C25%) demonstrated the ability to surpass the blocking effect caused by the precipitation of $C\bar{C}$ on the CH particles surface while producing a carbonated CPP similar that of the highest CO_2_ concentration (C80%). In fact, the high CO_2_ concentration and the corresponding quick and intense carbonation of CH produced significant alterations in carbonation conditions, namely, the temperature and water content of the medium. However, these conditions, essentially positive for the carbonation of C-S-H, did not counteract the aforementioned blocking effect of CH. The CO_2_ uptake difference between C40% and C25% was low (2.1%) at the end of the process. Yet, despite similar final outcomes, the CO_2_ concentration showed a significant impact on the CO_2_ uptake over time, especially in the first stage of the process where CH is the main component being carbonated. Thus, this work provides guidelines to adjust the minimum resident time required by the waste for a given CO_2_ concentration in an industrial context, aiming at the best compromise between process duration, CO_2_ uptake, and CO_2_ concentration. In fact, our results demonstrate that the recycled cement paste under the tested conditions is able to reach a CO_2_ uptake equivalent to that achieved after two weeks under atmospheric carbonation.

As expected, lowering the CPP maximum particle dimension increased the CO_2_ uptake. This influence was rather limited, however, with only a 1.3% increase in CO_2_ uptake when changing the particle dimension from 250 µm to 75 µm. Naturally, this analysis can shed light on the impact of this physical characteristic on CPP carbonation. However, its limited impact means that any possible consequences in the industrial process are unlikely, especially considering the increased energy costs usually associated with the grinding and sieving stages.

Regarding the influence of the CPP powder thickness, our results showed similar CO_2_ uptake within the 5 mm top layer for the different CO_2_ concentrations tested. However, carbonation was only able to proceed in depth at the highest CO_2_ concentration (80%), reaching 15 mm after 2 h.

Pure and impure CPP revealed similar results regarding the carbonation degree of the hydrated cement paste (CO_2_ uptake ranging from 15–20%). However, in terms of the total amount of powder under carbonation, the reduction in purity from 69% to 15% (cement proportion in weight) caused a decrease in CO_2_ uptake from 14 g to 2.8 g (mass of CO_2_ by mass of powder) due to the lower amount of hydrated cement paste present in the untreated material.

Hence, the key findings of this paper can be summarized as follows:

1. Adopting an initial water content in CPP between 16–21% provided an increase in the CO_2_ uptake that can reach 20%.
2. After two hours, the carbonation degree of CPP was similar for the different CO_2_ concentrations. While the higher CO_2_ concentration provided more intense C-S-H carbonation, this was counteracted by the shield effect of $C\bar{C}$, which prevented the complete carbonation of CH. However, the evolution of the CPP carbonation rate within the two-hour process indicates that the minimum resident time for maximum CO_2_ uptake decreases with increasing CO_2_ concentration.
3. The reduction of the maximum CPP particle dimension showed a limited increase in CO_2_ uptake, specifically, an increase of 1.3% for a size reduction from 250 µm to 75 µm.
4. The CO_2_ concentration had a positive impact on the carbonation depth, which may be relevant in industrial applications where the material under carbonation reaches higher quantities.
5. The short-term carbonation process was able to provide similar carbonation degrees of the hydrated cement paste regardless of the presence of aggregate impurities in the CPP powder.

Overall, this paper demonstrates that, among the parameters considered, the initial water content and the CO_2_ concentration in the carbonation chamber had the highest impact on the development of a more efficient and feasible carbonation process. The powder thickness revealed a significant impact as well, indicating that increasing the CO_2_ concentration is a viable solution when dealing with a high amount of CPP. Moreover, this paper further confirms previous findings that the low carbonation rate of C-S-H in the second stage of the process (where almost all CH has carbonated) is the limiting factor hindering higher carbonation of CPP.

Further work should focus on the development of a dynamic short carbonation process with cyclic variation of parameters, e.g., relative humidity or CO_2_ concentration, and on the development of methodologies that tackle the mechanisms hindering the low carbonation rate of C-S-H during the second phase of the process. Consequent improvements in the short duration carbonation process can lead to a feasible industrial process, and thus to the further valorisation of this concrete recycling byproduct.

## Figures and Tables

**Table 1:** Concrete composition.

Aggregate Type	w/c	OPC	Coarse Aggregate	Fine Aggregate	Water
Limestone gravel	0.55	360 kg/m^3^	406 L/m^3^	260 L/m^3^	198 L/m^3^

**Table 2:** Carbonation testing conditions and analytical techniques.

Testing Conditions Applied in Each Section	Testing Parameter Analysed in Each Section				
	3.1. IWC	3.2. CO_2_c	3.3. PS	3.4. LT	3.5. PT
**Initial water content (IWC)**	**≈0% to 30%**	15–20%	15–20%	15–20%	15–20%
**CO_2_ content (CO_2_c)**	80%	**25%, 40%, 80%**	80%, 25%	80%, 25%	80%, 25%
**Particle size (PS)**	250 µm	250 µm	**250, 75 µm**	250 µm	250 µm
**Layer thickness (LT)**	5 mm	5 mm	5 mm	**0–70 mm**	5 mm
**CPP powder type (PT)**	CPPp	CPPp	CPPp	CPPp	**CPPp, CPPc**
**Testing techniques**	IFM	TGA	IFM	IFM	IFM
**Stopping points**	3SPµ	1SP, 2SP, 3SP	2SP, 3SP	3SP	2SP, 3SP

## Data Availability

Data available on request due to restrictions.

## Abbreviations

1SP	First stopping point
2SP	Second stopping point
3SP	Third stopping point
ACC	Amorphous calcium carbonate
AFt	Ettringite
C25%	carbonation tests with a CO_2_ concentration of 25%
C40%	carbonation tests with a CO_2_ concentration of 40%
C80%	carbonation tests with a CO_2_ concentration of 80%
$C\bar{C}$	Calcium carbonate
CCUS	Carbon capture utilization and storage
CH	calcium hydroxide
CO_2_c	CO_2_ concentration parameter
CPP	cement paste powder
CPPc	CPP originated from concrete
CPPp	CPP originated from a cement paste
C-S-H	calcium silicate hydrate
IFM	Ignition by furnace method
IWC	Initial water content parameter
LOI	Loss on ignition
LT	Layer thickness parameter
PS	Particle size parameter
PT	CPP powder type parameter
RH	Relative humidity
SCM	supplementary cementitious materials
SPs	Stopping points
TGA	Thermogravimetric analysis
